# Comparison of Flow Reduction Efficacy of Nominal and Oversized Flow Diverters Using a Novel Measurement-assisted in Silico Method

**DOI:** 10.1007/s00062-024-01404-4

**Published:** 2024-04-23

**Authors:** Benjamin Csippa, Levente Sándor, Gábor Závodszky, István Szikora, György Paál

**Affiliations:** 1 Department of Hydrodynamic Systems, Faculty of Mechanical Engineering,, Budapest University of Technology and Economics, Műegyetem rkp 1–3, 1111 Budapest, Hungary; 2 National Institute of Mental Health, Neurology, and Neurosurgery, Department of Neurointerventions, Budapest, Hungary; 3 Faculty of Science, Informatics Institute, Computational Science Lab, University of Amsterdam, Amsterdam, The Netherlands

**Keywords:** Aneurysm, CFD, Hemodynamics, Stroke, Intervention

## Abstract

**Purpose:**

The high efficacy of flow diverters (FD) in the case of wide-neck aneurysms is well demonstrated, yet new challenges have arisen because of reported posttreatment failures and the growing number of new generation of devices. Our aim is to present a measurement-supported in silico workflow that automates the virtual deployment and subsequent hemodynamic analysis of FDs. In this work, the objective is to analyze the effects of FD deployment variability of two manufacturers on posttreatment flow reduction.

**Methods:**

The virtual deployment procedure is based on detailed mechanical calibration of the flow diverters, while the flow representation is based on hydrodynamic resistance (HR) measurements. Computational fluid dynamic simulations resulted in 5 untreated and 80 virtually treated scenarios, including 2 FD designs in nominal and oversized deployment states. The simulated aneurysmal velocity reduction (AMVR) is correlated with the HR values and deployment scenarios.

**Results:**

The linear HR coefficient and AMVR revealed a power-law relationship considering all 80 deployments. In nominal deployment scenarios, a significantly larger average AMVR was obtained (60.3%) for the 64-wire FDs than for 48-wire FDs (51.9%). In oversized deployments, the average AMVR was almost the same for 64-wire and 48-wire device types, 27.5% and 25.7%, respectively.

**Conclusion:**

The applicability of our numerical workflow was demonstrated, also in large-scale hemodynamic investigations. The study revealed a robust power-law relationship between a HR coefficient and AMVR. Furthermore, the 64 wire configurations in nominal sizing produced a significantly higher posttreatment flow reduction, replicating the results of other in vitro studies.

## Introduction

In the last decade flow diversion therapy for intracranial aneurysms has proved to be an efficient minimally invasive treatment technique. Functional reconstruction of the parent artery can be induced by implanting a densely woven mesh across the aneurysm neck that imposes a hydrodynamic resistance [1]. The resulting flow reduction promotes safe thrombosis inside the aneurysm sac. The PUFs trial [2] demonstrated high occlusion and low complication rates for the pipeline embolization device (PED), leading to the first FDA approval of flow diverters. Since then other devices have emerged in flow diversion therapy, and further clinical trials proved their high efficacy and low complication rates [3–5]. Yet only a few have both FDA and EMA approval due to the challenges of lengthy clinical trials. The outcomes of such clinical trials are commonly assessed by defining an endpoint that can be quantified in the context of the involved treatment. For the endovascular treatment of intracranial aneurysms (IA), the widely used clinical endpoint is the angiographic occlusion rate, measured after 6 and 12 months using the Raymond-Roy occlusion classification.

Computational fluid dynamics (CFD) is well-established in hemodynamics investigations of IAs [6–8]. Treatment methods were studied promptly [9] when initial animal experiments demonstrated promising results [10]. Multiple studies concluded [11–14] that aneurysmal mean velocity reduction (AMVR) might be a suitable surrogate in silico endpoint for the clinical endpoint, the occlusion of the aneurysm sac. The AMVR can be obtained from high-fidelity numerical computations. A retrospective clinical study by Ouared et al. [15] demonstrated that a numerically obtained threshold of 35% AMVR results in aneurysm occlusion with a 99% specificity. Recently, Frangi et al. [16] have given the first example of an in silico trial (IST) for IA treatment. The investigation of 82 virtual patients found a good match in flow diversion efficacy compared to previous PED-related clinical trials using AMVR as a surrogate endpoint. Furthermore, the authors showed the additional capability of ISTs by investigating the effect of hypertension, something that could not be done within a clinical trial. Even though AMVR shows significant promise, further investigation is necessary to establish it as a clinically relevant endpoint surrogate.

The aim of this paper is twofold. First, to present a workflow capable of scaling up case numbers to clinical trial standards that is more reproducible than previous manual methods. The impact of sizing FD devices on the occlusion rates were previously studied [17], yet a direct comparison of different manufacturers in this respect has yet to be analyzed. The second objective is to utilize this workflow in investigating the effect of the different deployment scenarios (nominal and oversizing) on the aneurysmal flow field of two FD types (48-wire PED, 64-wire P64), particularly on the posttreatment AMVR.

## Methods

**Fig. 1:**
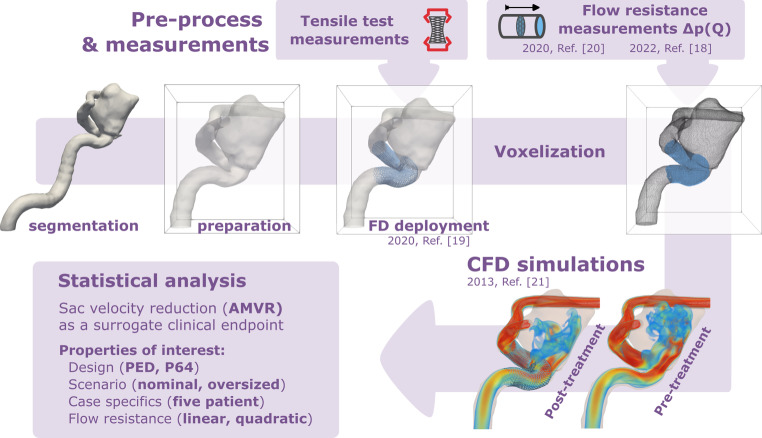
Framework for the virtual deployment and analysis of treatments. Previously created geometries are prepared for later lattice Boltzmann method (LBM) simulations. A calibrated mass-spring-damper model calculates the FD (Flow Diverter) deployment. The vessel and stent geometries are voxelized to be used in the automated LBM simulations. Post-treatment aneurysmal mean flow reduction (AMVR) is evaluated and gathered for all cases to be used in the statistical analysis

Internal carotid artery (ICA) aneurysms of five patients who underwent endovascular treatment were selected for this study. The patients were treated with either FDs or FD-assisted coiling and clinical follow-up showed complete occlusion. Medical images from 3D DSA angiography were acquired using a GE Innova IGS 630 system (GE HealthCare Technologies, Inc., Chicago, Illinois, USA). The internal review board of the hospital approved the study, and all patients gave written consent. First, the medical images were segmented using Slicer 4.11 (Kitware Inc., New York, NY, USA). The resulting vessel surfaces were further processed and smoothed in MeshLab (ISTI-CNR, Visual Computing Lab) and the Vascular Modeling Toolkit (Orobix Inc). A measurement-supported numerical workflow was developed (as shown in Fig. 1) to streamline and automate the virtual deployment and the hemodynamic simulation. The following subsections briefly describe the measurement and modelling principles (more details can be found in [18–21]). The methodological differences compared to other manual workflows are summarized in the “Discussion” section.

### Flow Diverter Deployment

The virtual deployment of a flow diverter is simulated using an interconnected spring network model that mimics the braided structure of an FD device [19]. The mechanics of the stent is modelled by two different linear spring types with varying coefficients of stiffness. One type, the surface spring, is responsible for replicating the length contraction, while the other type, the radial spring, models the radial deformation due to length contraction or elongation. The stiffness coefficients are set according to a detailed force-response optimization, based on data from mechanical tensile testing measurements. A graphical user interface was developed to visually inspect the FD deployment into the geometry. The simulation models the complete release process of the flow diverter from the sheathing catheter. The resulting stent surface is saved and transferred to the subsequent flow simulations (see Fig. 1).

### Porous FD Approach Based on Flow Resistance Measurements

Currently, two distinct methodological approaches are in use for incorporating FDs into a CFD simulation: (i) representing the wires with fully resolved geometry; (ii) representing the effective flow resistance of the FD surface by a porous layer. Both of these approaches have advantages and disadvantages. The current work is based on the second approach. The main difference lies in the concepts of the underlying approximations. A fully resolved FD based on a mathematical deployment model approximates the wire positions and angles of a specific deployment; however, the exact positions and the specific strut structure of that approximated deployment are unavailable as the precision of clinical imaging modalities is about 200 microns and the wire diameter of FDs is 30 microns. A homogeneous porous layer approach describes a measurement-based approximate deployment without any further assumptions. Another difference is the computational cost. Although the direct approach is more detailed, the inherent numerical meshing constraints imply a significantly higher computational cost. Hence, in our workflow to scale up future in silico studies, the flow diverters are currently modelled by a homogeneous porous layer. The porous layer of the FD model is incorporated into the fluid dynamical equations via the Darcy-Forchheimer law. This model describes the flow resistance of the FD as a function of a linear and a quadratic resistance coefficient. A purpose-built measurement setup was developed previously to obtain these quantities from FD samples [20].

Recently, FDs from two manufacturers were measured under different deployment conditions and a comparison between resistance and metallic surface area (MSA) was made [18]. The FDs were implanted into the measurement device by an experienced neurointerventionist. The FD insertions were repeated several times for each scenario to capture the variability in the deployment procedure and in the resulting measured hydrodynamic resistance. The 4‑mm and 5‑mm FDs were implanted into a 4-mm holder tube to investigate nominal and oversized scenarios.

The pressure drop on the stent surface as the function of the flow rate was measured as described in [20] to obtain the linear and quadratic coefficients. The known outflow area in the measurements was used to calculate these coefficients from the expression of the Darcy-Forchheimer law, which describes the pressure drop across a porous layer:

1 $$\Updelta p=l_{c}*v+q_{c}*v^{2},$$

where l_c_ and q_c_ represent the linear and quadratic hydrodynamics resistance factors, respectively, v is the average velocity at the ostium of the artificial vessel from the measurement.

Data used in this study are summarized in Table 1. The 80 scenarios include data from 2 manufacturers (Pipeline Embolization Device, Medtronic, Minneapolis, MN, USA: referred to as PED; P64 flow modulation device, Phenox, Bochum, Germany: referred to as P64) in different deployment conditions. Nominal deployment represents the case when the diameter of the FD matches the diameter of the artificial vessel in our measurement setup, while “oversized” indicates that the artificial vessel diameter is 1 mm smaller than the FD diameter.

**Table 1:** Measured hydrodynamic resistance (HR) coefficients (l_C_ [kg/m^2^s] and q_C_ [kg/m^3^]) for all 16 considered deployment scenarios

		PED								P64							
		1	2	3	4	5	6	7	8	9	10	11	12	13	14	15	16
Nominal	l_C_	66.8	114	137	135	–	–	–	–	186	164	160	–	–	–	–	–
	q_C_	170	464	133	131	–	–	–	–	139	187	349	–	–	–	–	–
Oversized	l_C_	–	–	–	–	5.6	4.3	4.7	42.8	–	–	–	5.8	19.3	6.3	9.5	36.1
	q_C_	–	–	–	–	239	281	267	157	–	–	–	316	232	308	243	204

### Simulation Description

The hemodynamic equations were solved using the lattice Boltzmann method (LBM) with an in-house code [21]. The encompassing framework includes three steps. In the first step, the geometry is prepared for simulation, including the voxelization of the mesh and the treatment of the boundaries. The vessel segments are automatically extended by five diameters in length to neutralize the effect of boundaries [22].

Transient blood flow simulations were executed to compute one heart rate cycle, after a precursor warm-up phase to the 0th time-step conditions [21], with a time-varying inlet waveform [12] and parabolic velocity profile. The cross-sectional average inlet velocity was calculated based on the inlet area, following the method of Cebral et al. [23], multiplied by the reciprocal value of the waveform cycle average. The outlets were set according to Murray’s law [24] and 0 Pa relative pressure was imposed on the outlet with the smallest diameter. The vessel wall was assumed to be rigid. Blood was considered an incompressible fluid with Newtonian rheology with a density of 1055 kg/m^3^ and a dynamic viscosity of 3.4 mPa s. The voxel space contained approximately 5 million lattices, resulting in an average spatial resolution of 0.1 mm and time-step values in the order of 10^−4^ s. For this study 50 time steps were exported during the heart cycle to calculate the cycle-averaged quantities.

### Evaluation Techniques and Study Design

A neck detection algorithm was developed based on Piccinelli’s [25] method to calculate the ostium plane to separate the aneurysm sac. Space and time-averaged velocity (STAV) for the aneurysm was computed for the base and posttreatment cases. The aneurysm mean velocity reduction (AMVR) was calculated for all post-flow diversion cases as follows:

2 $$AMVR=\frac{\mathrm{STAV}_{\mathrm{pre}}-\mathrm{STAV}_{FD}}{\mathrm{STAV}_{\mathrm{pre}}}$$

where the subscripts *pre* and *FD* stand for the pretreatment and post-FD treatment simulations, respectively.

**Fig. 2:**
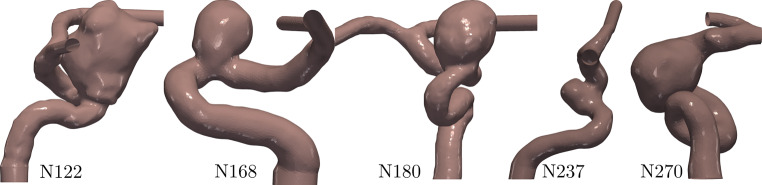
Utilized patient-specific geometries of intracranial aneurysms in the study

This study evaluated the flow response to different flow diverter deployments, five patient-specific cases were selected, as depicted in Fig. 2, and the following deployments were considered (also listed in Table 1): four PED and three P64 deployments were nominal, while four PED and five P64 cases were considered for the oversized cases. In total, five untreated and 80 posttreatment simulations were studied (summarized in Table 2).

**Table 2:** Study characteristics of the simulation case study based on the various deployment scenarios

PED		P64		Treated	Pre	Total
Nominal	Oversized	Nominal	Oversized			
4	4	3	5	80	5	85

## Results

Virtual deployments of flow diverters were performed for five virtual patients. Both manufacturers were considered with 4‑mm and 5‑mm diameter devices (nominal and oversized scenarios, respectively) with their corresponding force-response calibrated mechanistic models. The simulations were constructed and run on the Snellius HPC cluster (SURF, Netherlands).

### Flow Resistance

**Fig. 3:**
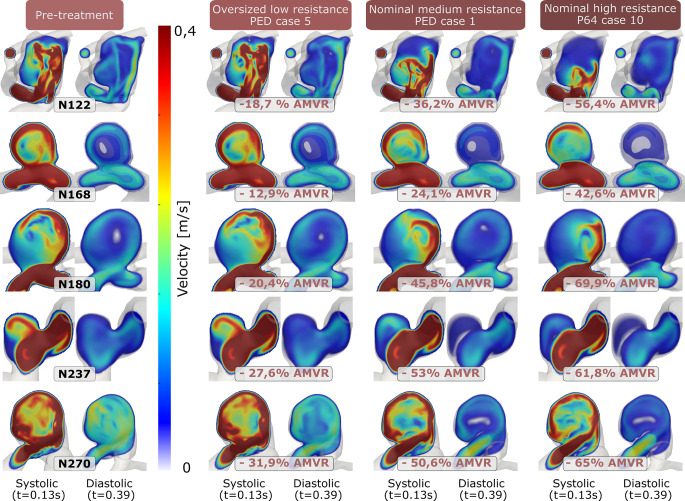
Visualization of the flow field in systolic and diastolic time instants with increasing hydrodynamic resistance. Columns from *left to right*: pretreatment simulation; low resistance (P64, oversized); medium resistance (PED, nominal); high resistance (P64, nominal)

Figure 3 demonstrates cross-sections of the aneurysmal flow fields for the pretreatment state and three other cases with increasing levels of resistance corresponding to the low, medium, and higher ranges of the resistance spectrum. For each case, the systolic and diastolic time instances are shown. At low resistance, corresponding to an oversized P64 deployment (case 12), all geometries display only a minor velocity reduction. In the medium resistance cases of a nominal PED deployment (case 1, third column in Fig. 3), the observed decrease in the velocity is more prominent. The systolic and diastolic velocity snapshots show that the inflow jet break up is more intense with the increase of hydrodynamic resistance. Furthermore, the N237 case demonstrates a qualitative flow pattern change due to flow redirection. Finally, the high resistance cases of the nominal deployments (P64, case 10) display further velocity decrease while an almost vanishing jet flow in the diastolic instant.

**Fig. 4:**
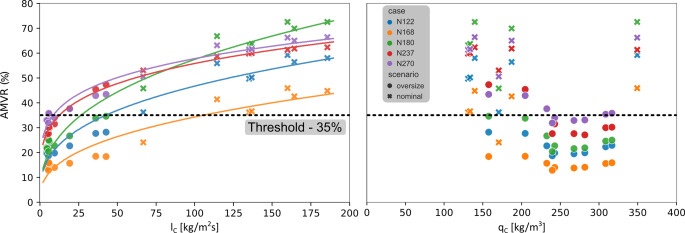
Velocity reduction as a function of the linear – l_C_ (*left*) and quadratic – q_C_ resistance coefficient (*right*) incorporating all deployment scenarios. The *black dashed line* represents the 35% velocity reduction threshold value

The quantitative relationship between the two resistance coefficients and the resulting AMVR was investigated. The quadratic coefficient showed no consistent functional relationship with AMVR, as opposed to the linear coefficient (Fig. 4b). Figure 4a shows the dependence of AMVR on the linear resistance coefficient, l_c_. A strong power law relationship can be identified with the lowest coefficient of determination of 0.91 for all patient-specific anatomies, considering all deployment scenarios and FDs without any classification. Furthermore, the figure also shows that oversized scenarios have significantly smaller linear resistance values and induce a lower velocity reduction than the nominal cases.

### Wire Number

**Fig. 5:**
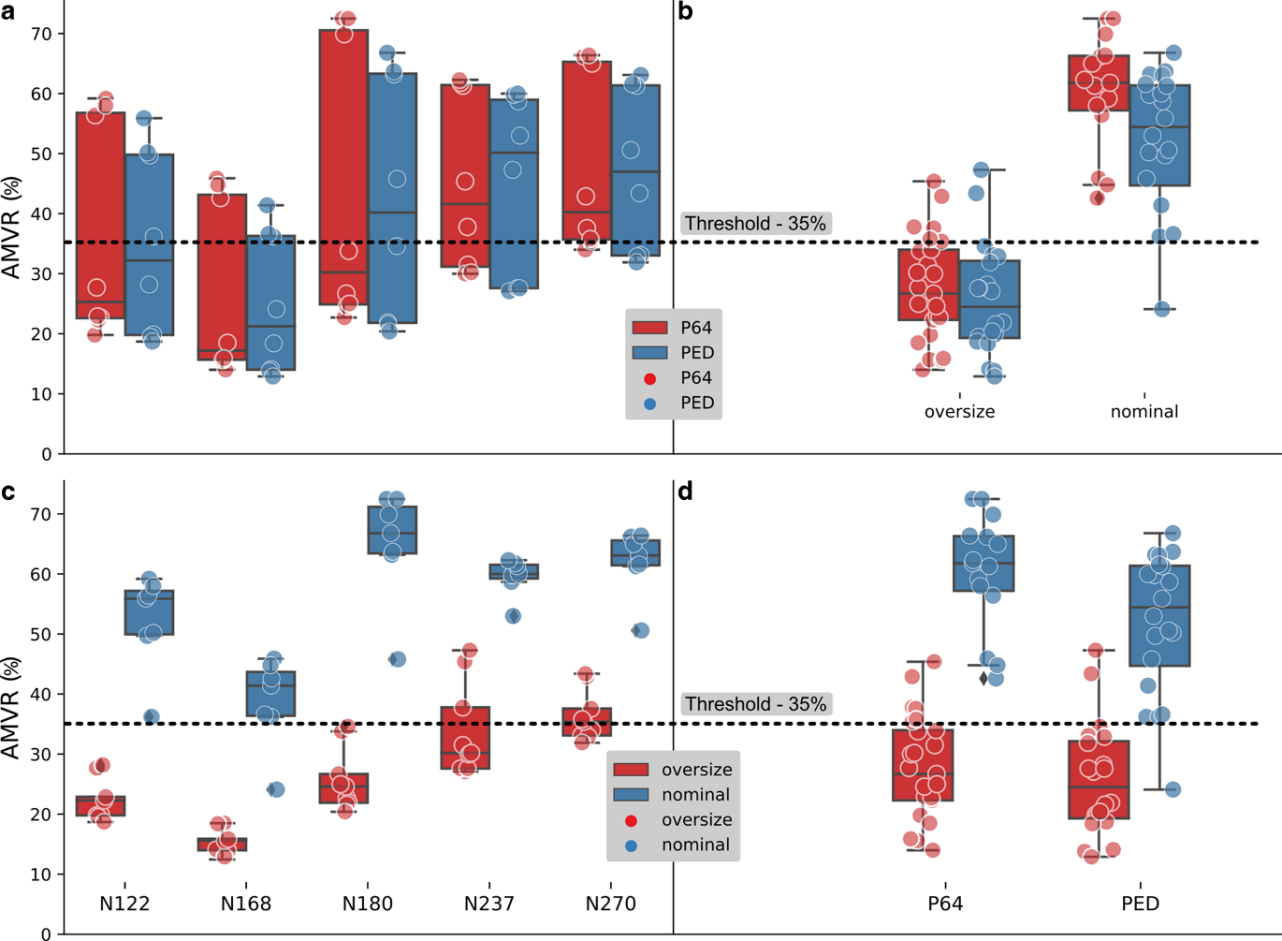
Boxplots with associated data points for comparing different group classifications. The *black dashed line* represents the 35% velocity reduction threshold value. **a** Patient-specific and manufacturer classification. **b** Deployment and **d** manufacturer classification. **c** Patient-specific and deployment classification. The color definition of the **a**-**b** panels change from the manufacturers to deployment scenario for the **c**-**d** panels

Quantitative results are shown in Table 3 for each patient. In general, effective flow diversion can be achieved for all studied patients, considering a 35% AMVR as a surrogate clinical endpoint. Other than the N168 case, the average AMVR obtained for both PED and P64 cases for each patient-specific geometry reached the level of threshold but with different degrees of scattering (see Fig. 5a). The lowest AMVR were obtained for the N168 case and only the higher nominal scenarios generated flow reductions above the threshold (Fig. 5a). The largest AMVR was obtained by a P64 scenario in the case of N180 (72.5%) while the highest average was reached in the case of N270 (47.6%).

**Table 3:** Summary of the results for all patients by different classifications of the deployment scenarios. Each row represents an average AMVR (%) value and its bounds in parentheses. In order from top to bottom: all deployments (SUM); only PED scenarios; only P64 scenarios; only nominal scenarios; only oversized scenarios

	N122	N168	N180	N237	N270
SUM	35.5 (18.7–59.2)	25.6 (12.9–45.9)	42.8 (20.4–72.5)	45.1 (27.1–62.3)	47.6 (31.9–66.4)
PED	34.8 (18.7–55.9)	24.7 (12.9–41.4)	42.2 (20.4–66.8)	45.1 (27.1–60)	47.2 (31.9–63.1)
P64	36.1 (19.8–59.2)	26.6 (14.0–45.9)	43.4 (22.7–72.5)	45 (30–62.3)	47.9 (34.0–66.4)
Nominal	52.2 (36.2–59.2)	38.8 (24.1–45.9)	64.9 (45.8–72.5)	59.5 (53.0–62.3)	62 (50.6–66.4)
Oversized	22.4 (18.7–28.2)	15.4 (12.9–18.5)	25.7 (20.4–34.6)	33.8 (27.1–47.3)	36.3 (31.9–43.4)

### Deployment Scenario

The patient by patient outcome of classifying the scenarios into nominal and oversized categories is shown in Fig. 5c and reported in Table 3. It is apparent that oversizing the device by 1 mm in this cohort of investigated scenarios does not yield a satisfactory outcome, according to the chosen endpoint; however, in two cases (N237, N270), some deployment scenarios reach the threshold level of 35% AMVR. Table 3 shows that the average AMVR of the nominal scenarios is above the 35% threshold, the lowest being 38.8% for the N168 case. Furthermore, other than a few scenarios in the case of N168, the AMVR of almost all nominal scenarios exceeds the threshold.

### Cumulative Results

The combined results are shown in Fig. 5b, d. Both panels include the same boxplots but with different classifications. The result of this classification is summarized in Table 4 with the average values and their standard deviation. The results in Fig. 5b demonstrate again that oversizing yields unfavorable outcomes. Additionally, the average STAV reductions of the two FD types in this deployment condition are almost the same (25.7% and 27.5%); however, a significant difference (*p* < 0.0258) was observed between the two FD types for the nominal deployments. The P64 scenarios displayed a larger average AMVR (60.3%) than the PED scenarios (51.9%).

**Table 4:** Summary of the cohort average AMVR by different classifications and their standard deviations

	PED	P64	p
*Nominal*	**51.9 (11.8)**	**60.3 (9.5)**	**<** **0.0258**
*Oversized*	25.7 (9.4)	27.5 (8.8)	0.517

Last column: level of significance on the comparison of means of the stent types

## Discussion

### Flow Resistance

The investigated FD deployment scenarios revealed a power-law relationship between the measured linear resistance coefficient and AMVR (see Fig. 4a, fitted curves). As any FD and deployment scenario can be represented with a porous FD model, the relationship described above is valid for any woven stent type and depends only on the HR. A deeper insight into the dependence of the parameters of the power-law relationship and the patient’s anatomy cannot be obtained from the current results due to the small number of investigated patients. Presumably, however, geometric factors of the aneurysm sac and the local morphological features of the parent vessel could be influential determinants. Furthermore, the quadratic HR coefficient does not have a functional relationship with AMVR but can increase the AMVR on a smaller scale, primarily in oversized situations when the linear HR coefficient is small. Regarding future in silico trials, the calculation of this power-law relationship could be helpful in the quantification of the deployment scenarios of new FD designs.

### FD Design and Deployment

A significantly higher average flow reduction (+8.4% AMVR) was obtained in nominal scenarios for P64 cases. This result was expected as, in our previous work [18], we have shown that the average MSA of the nominal 64-wire configurations is higher (MSA = 0.42) compared to the 48-wire nominal configurations (MSA = 0.34). Similar in vitro results were obtained for the effect of wire numbers by [26] and [27], which may explain the industrywide tendency to design higher wire-count FDs [28]. Oversizing FDs is known to have a decreasing effect on the flow reduction capacity [17]. In our case study, the relative decrease for PED is around 50% and about 54% for P64; however, surprisingly, in the oversized scenario the average flow reduction capacity of 64-wire and 48-wire FDs were statistically very similar. Although further evidence is necessary, the clinical interpretation of this result raises a methodological question: “does oversizing with a large wire count FD to establish better wall apposition still yield sufficient flow reduction?”

### General Discussion

As opposed to the constant radius measurement device, real vessels are tapered towards the distal direction, and FDs are selected to ensure proper apposition, especially at the aneurysm neck, which implies that a usual device selection is always slightly oversized. Furthermore, in the present study, FDs were modelled as homogeneous porous layers, not incorporating the effects of “push-pull” compression during the deployment. Thus, from a clinical perspective the nominal and oversized deployment scenarios investigated here represent the opposite ends of clinical sizing considerations. This argument can be further confirmed by collecting the MSA values from the precursor measurement study [18]. The average MSAs in nominal sizing were 42% and 34% and 26% and 21% in oversizing for the 64 and 48 wire scenarios, respectively. According to [29] 70% porosity or 30% MSA is sufficient for aneurysm occlusion. Thus, the MSAs of the reported scenarios in [18] are 12% and 4% higher for the nominal and 4% and 9% smaller in oversized 64-wire and 48-wire configurations, respectively.

Computational modelling and simulation (CM&S) is widely used in FD research; with simulation-based tools optimization of the procedure or new designs of such devices can be achieved [30–32]. Recently, these technologies found their way into clinical practice as in silico technologies with applications for preoperative planning and selection of devices by simulating the virtual deployment of FDs. The FDA has already approved some of these applications as a clinical decision support system [33, 34]. An even more promising solution in the near future is in silico (clinical) trials (IST), which refers to the use of computer simulations in the development or regulatory assessment of new products (medicines or devices) and medical procedures. Viceconti et al. [35] presented the concept of IST and what is needed for such solutions to qualify as a credibility assessment tool for new products. Such a qualification protocol has recently been established by the American Society of Mechanical Engineers (ASME) and has been recommended to be used by the FDA in the ASME VV-40-2018 standard and in the EU, a community of specialists has already proposed a possible pathway for the CE marking system [36]. The abovementioned research studies paved the way for the initial design of IST for aneurysm flow diverter treatments.

The porous layer approach, when properly used, has been recognized as a sufficiently accurate technique for CFD simulations of FD treatment with a nonnegligible advantage of lower computation time and costs. Compared to former CFD workflows, the core novelty of the present one is the inclusion of mechanical tensile testing and hydrodynamic resistance measurements to obtain device-specific data for the deployment and porous layer model, respectively. The combination of in vitro measurements and in silico methods offers new insights [16] and opportunities to quantify numerical FD treatment, such as the variations in deployment scenarios. Furthermore, applications like the one reported here can speed up computations and scale up the number of simulations to the standards of formal clinical trials.

### Limitations

The current study employs a relatively small cohort of five patient-specific geometries. The investigation was done retrospectively as the objective was to investigate the effect of deployment variability. The CFD simulations adopted assumptions often used in similar works. In particular, the blood was assumed to be a Newtonian fluid, and the vessel walls were considered to be rigid. A population-based average waveform was scaled patient-specifically to impose inflow boundary conditions. Further assumptions were made for the numerical FD model. The porous layer was assumed to be homogeneous and isotropic. Nonhomogeneous characterization of the FDs is currently under implementation in our model by mapping the HR measurement data onto the virtually deployed device when an adequate amount of HR data will be measured. Clinical deployment techniques, like the push-pull method, were not incorporated although the capability is present in our virtual deployment model.

## Conclusion

A measurement-supported numerical framework was presented for the virtual deployment and hemodynamic analysis of flow diverter devices. The presented application was purposefully built with imminent in silico trials in mind.

A strong power-law relationship was found between the linear HR coefficient and AMVR. This functional relationship could quantify the flow reduction capacity of clinically used deployment procedures for a given patient. In nominal deployment scenarios, the FDs with higher wire numbers caused a larger AMVR, similar to that reported in previous in vitro studies [26, 27]; however, somewhat surprisingly, the average flow reductions in oversized deployments were the almost same for 48-wire and 64-wire configurations.

## Data Availability

Not applicable
